# Hydrolyzed Rice Formula for the Management of Cow’s Milk Allergy in Infants and Young Children: A Systematic Review of Randomized Controlled Trials

**DOI:** 10.3390/nu18203429

**Published:** 2026-10-19

**Authors:** Hania Szajewska, Elżbieta Jarocka-Cyrta, Agata Stróżyk, Andrea Horvath, Urszula Jedynak-Wąsowicz, Anna Liber, Joanna Rachtan-Janicka, Jarosław Kwiecień

**Affiliations:** 1 Department of Paediatrics, Medical University of Warsaw, 02-091 Warsaw, Poland; agata.strozyk@wum.edu.pl (A.S.); andrea.horvath@wum.edu.pl (A.H.); 2 Department of Pediatrics, Gastroenterology and Nutrition, University of Warmia and Mazury, 10-719 Olsztyn, Poland; ejarocka@op.pl; 3 Department of Pediatrics, Jagiellonian University Medical College, 31-007 Krakow, Poland; ula.jedynak-wasowicz@uj.edu.pl; 4 Department of Nutrition, Institute of Mother and Child, 01-211 Warsaw, Poland; anna.liber@imid.med.pl; 5 Department of Functional and Organic Food, Warsaw University of Life Sciences, 02-787 Warsaw, Poland; joanna_rachtan-janicka@sggw.edu.pl; 6 Department of Pediatrics, Faculty of Medical Sciences in Zabrze, Medical University of Silesia in Katowice, 40-555 Zabrze, Poland; jkwiecien@sum.edu.pl

**Keywords:** cow’s milk allergy, hydrolyzed rice formula, infants, tolerance acquisition, growth, systematic review

## Abstract

**Background/Objectives**: Hydrolyzed rice formula (HRF) is an alternative to extensively hydrolyzed cow’s milk formula (eHF) or soy formula for infants with cow’s milk allergy (CMA). We evaluated its effects on acquisition of tolerance to cow’s milk protein, growth, safety, and tolerability in infants and young children with confirmed CMA. **Methods**: PubMed, Embase, and CENTRAL were searched through 26 May 2026 without language restrictions (PROSPERO CRD420261445267). Risk of bias was assessed using the Cochrane Risk of Bias 2 tool for tolerance acquisition and growth, and certainty of evidence was assessed using the Grading of Recommendations Assessment, Development and Evaluation (GRADE) for tolerance acquisition. Owing to heterogeneity, results were synthesized narratively. **Results**: Four RCTs (321 participants; range, 16–108) were included. Two reported tolerance acquisition, assessed at 12 months of age in one trial and during 12 months of follow-up in the other. Estimates were imprecise and in opposite directions: RR 0.84 (95% CI 0.53–1.32; 18/41 vs. 21/40) and RR 1.35 (95% CI 0.95–1.91; 26/36 vs. 22/41). Certainty of evidence was very low. Growth did not differ significantly between HRF and eHF in two trials. In another trial, an unadjusted advantage for HRF and casein hydrolysate over soy formula did not persist after correction for multiple comparisons. Allergic reactions were rare, with no clear differences in adverse events between formulas. **Conclusions:** Available evidence does not establish whether HRF is equivalent, superior, or inferior to eHF or soy formula for tolerance acquisition or growth in infants with CMA. Adequately powered RCTs using standardized outcome definitions are needed.

## 1. Introduction

### 1.1. Description of the Condition

Cow’s milk allergy (CMA) is one of the most common food allergies in infancy and early childhood. Management is based on eliminating cow’s milk protein while ensuring adequate nutrition and normal growth. Breastfeeding should be continued whenever possible. When breastfeeding is insufficient or not possible, specialized formulas such as cow’s milk-based extensively hydrolyzed formula (eHF) or amino acid formula (AAF) are an essential component of dietary management [1].

### 1.2. Description of the Intervention

Hydrolyzed rice formula (HRF) is a plant-based alternative to formulas derived from cow’s milk protein. Rice protein undergoes enzymatic hydrolysis to improve its water solubility. Its amino acid composition differs from cow’s milk protein, with lysine and threonine among the limiting amino acids. HRFs are therefore supplemented with selected amino acids to meet the amino acid requirements of infants and young children. HRFs vary in protein content, degree of hydrolysis, and peptide size distribution. Moreover, molecular weight profiles are reported using different metrics that are not directly comparable across products [2,3].

Concerns have been raised about the arsenic exposure from rice-based products. Available evidence suggests that arsenic exposure from HRF may be comparable to that from cow’s milk-based formula [4,5]. However, these findings do not establish the safety of arsenic exposure from HRF.

HRF is also generally reported to have better palatability than eHF or AAF [5], although comparative evidence on acceptance and adherence remains limited.

HRFs incorporating additional components such as biotechnologically manufactured human milk oligosaccharides are now being evaluated in clinical trials. A single-arm study assessing the tolerance and growth of an HRF containing 2’-fucosyllactose (2’-FL) alone has been published [6]. In addition, two study protocols evaluating HRF containing both 2’-FL and lacto-N-neotetraose (LNnT) have been published, although results are not yet available [7,8].

### 1.3. How the Intervention Might Work

Unlike cow’s milk-based eHF, HRF contains no cow’s milk protein and therefore avoids exposure to residual cow’s milk peptides, which may cause reactions in a small proportion of children with CMA. This has been proposed as a potential advantage of HRF, particularly in children who do not tolerate eHF [9]. Whether HRF influences the acquisition of tolerance to cow’s milk protein remains uncertain, and the underlying mechanisms are poorly understood [2].

### 1.4. Rationale for the Review

A previous systematic review (search up to May 2024) of HRF for the management of CMA included heterogeneous study designs, such as non-randomized studies, and heterogeneous populations, such as healthy infants without CMA [3]. These limitations reduce the certainty and applicability of their conclusions to infants and young children with confirmed CMA. An updated review should therefore focus on randomized evidence in infants and young children with confirmed CMA, incorporate more recent trials, and keep clinically distinct outcomes separate.

In particular, efficacy and tolerability in managing allergic symptoms, effects on growth and nutritional adequacy, and the acquisition of tolerance to cow’s milk protein represent distinct clinical questions and should not be combined into a single overall assessment of efficacy. An updated, methodologically rigorous systematic review addressing these issues is therefore warranted.

This systematic review was undertaken specifically to inform a position paper developed by the Food Allergy Section and the Nutrition Section of the Polish Society for Paediatric Gastroenterology, Hepatology and Nutrition. Its findings may also inform other guidelines and recommendations on the dietary management of CMA.

### 1.5. Objectives

The primary objective was to evaluate the effect of HRF on the acquisition of tolerance to cow’s milk protein in infants and young children with confirmed CMA. The secondary objectives were to evaluate: (1) growth and nutritional outcomes; and (2) safety, tolerability, and allergic outcomes associated with the use of HRF for the dietary management of confirmed CMA.

## 2. Materials and Methods

The protocol was registered in PROSPERO (CRD420261445267). The review was conducted and reported in accordance with the Preferred Reporting Items for Systematic Reviews and Meta-Analyses (PRISMA) [10] statement and methodological guidance from the Cochrane Handbook for Systematic Reviews of Interventions [11].

### 2.1. Eligibility Criteria

#### 2.1.1. Types of Studies

We included randomized controlled trials (RCTs) evaluating HRF for the dietary management of confirmed CMA in infants and young children. Non-randomized and quasi-randomized studies, case reports, case series without a comparator, narrative reviews, editorials, letters without usable original data, and animal or in vitro studies were excluded.

#### 2.1.2. Types of Participants

We included infants and young children with confirmed CMA (according to the definition used by the study authors), including IgE-mediated CMA, non-IgE-mediated CMA, and mixed CMA populations, provided that the diagnosis of CMA was explicit and relevant data could be extracted. We recorded the diagnostic method, including oral food challenge (OFC), elimination and reintroduction, specific IgE testing, skin prick testing, clinical history, or physician diagnosis.

Eligible participants were aged 0–36 months. Studies with a slightly wider upper age range were not excluded solely on this basis if the study population consisted predominantly of infants and young children and formula treatment was clinically relevant.

We excluded prevention studies in children without established CMA; studies evaluating general feeding tolerance without a clear diagnosis of CMA; studies of plant-based beverages or other non-formula rice products; and studies in which HRF was mentioned but was not clearly evaluated as an intervention.

#### 2.1.3. Types of Interventions

The intervention of interest was HRF used for the dietary management of CMA. We included any infant formula based on hydrolyzed rice protein, regardless of the reported degree of hydrolysis. HRF containing additional components, including biotics or other functional ingredients, was eligible provided that hydrolyzed rice protein constituted the protein base of the formula. Eligible comparators included specialized formulas used to manage CMA, such as eHF, AAF, and soy formula.

### 2.2. Outcome Measures

Outcome measures were prespecified based on those evaluated in the World Allergy Organization Diagnosis and Rationale for Action against Cow’s Milk Allergy (DRACMA) systematic review [12].

#### 2.2.1. Primary Outcome

The primary outcome was acquisition of tolerance to cow’s milk proteins (as defined by the study authors). We recorded the method used to establish tolerance, including whether tolerance was confirmed by OFC. Studies using alternative definitions were retained, and differences in outcome definition were considered when assessing indirectness and certainty of the evidence. Tolerance acquisition was extracted at all reported time points, particularly at 6, 12, 18, and 24 months, where available.

#### 2.2.2. Secondary Outcomes

Growth and nutritional outcomes included growth parameters, such as weight, length/height, weight-for-length or body mass index, head circumference, and corresponding Z-scores, and failure to thrive. Safety, tolerability, and allergic outcomes included clinical allergic reactions to the administered formula, vomiting, diarrhea, urticaria, eczema, recurrent wheezing, asthma exacerbations or other respiratory allergic reactions, epinephrine use, development of food protein-induced enterocolitis syndrome (FPIES), adverse events, change or discontinuation of the assigned formula because of adverse effects, and sensitization to the administered formula. Sensitization was extracted according to the definition used by the study authors. Where possible, we distinguished immunological sensitization, such as a positive skin prick test or formula-specific IgE, from clinical allergic reactions to the administered formula. Studies including mixed IgE-mediated and non-IgE-mediated populations were eligible. When phenotype-specific data could not be extracted, results were interpreted with caution because of potential clinical heterogeneity and indirectness.

### 2.3. Search Methods

#### 2.3.1. Electronic Searches

We systematically searched MEDLINE (via PubMed), Embase, and the Cochrane Central Register of Controlled Trials (CENTRAL) from database inception to 26 May 2026. We also searched ClinicalTrials.gov and the WHO International Clinical Trials Registry Platform to identify ongoing, completed, or unpublished trials. No language restrictions were applied. The complete search strategies are provided in Table S1.

#### 2.3.2. Searching Other Resources

Reference lists of eligible studies and previous systematic reviews were screened for additional potentially eligible studies. Experts in the field were contacted to identify additional or unpublished studies. Conference abstracts and proceedings without sufficient information to permit assessment of eligibility and extraction of relevant outcome data were excluded.

### 2.4. Data Collection and Analysis

#### 2.4.1. Selection of Studies

Three reviewers (AS, AL, UJW) independently screened titles and abstracts identified by the searches. Full texts of potentially eligible reports were subsequently assessed independently against the predefined eligibility criteria. Disagreements were resolved through discussion or, when necessary, consultation with a third reviewer. Multiple reports relating to the same study were linked and treated as a single study. The study selection process was documented using a PRISMA flow diagram (Figure S1), and reasons for exclusion at the full-text stage were recorded.

#### 2.4.2. Data Extraction and Management

Two reviewers (AH, EJC) independently extracted data using a standardized data extraction form. Disagreements were resolved through discussion or consultation with another reviewer. For each included study, we extracted study characteristics (country, setting, recruitment period, study design, sample size, funding, conflicts of interest, trial registration, and duration of follow-up); participant characteristics (age, sex, CMA phenotype and diagnostic criteria, baseline severity, relevant co-allergies, nutritional status, and eligibility criteria); and detailed characteristics of the intervention and comparator. For HRF, these included the product and manufacturer, protein source and content, degree of hydrolysis and peptide-size distribution, formula composition, amino acid supplementation, additional components, dose or feeding regimen, duration, adherence, and co-interventions. For each outcome, we extracted its definition, time point, analysis population, number of participants, numerical outcome data, reported effect estimates, and relevant safety data.

#### 2.4.3. Assessment of Risk of Bias

Three reviewers (UJW, JK, AS) independently assessed risk of bias using the Cochrane Risk of Bias 2 (RoB 2) tool [13]. Disagreements were resolved by discussion or consultation with another reviewer. The following five domains were assessed: bias arising from the randomization process; bias due to deviations from intended interventions; bias due to missing outcome data; bias in measurement of the outcome; and bias in selection of the reported result.

Risk of bias was assessed in full for the primary outcome (acquisition of tolerance) and for growth, a secondary outcome of particular clinical relevance to this review. Because not all included trials reported both outcomes, each assessment was restricted to the trials contributing data: two trials for tolerance acquisition [14,15] and four trials for growth [14,15,16,17]. Risk of bias was categorized as low risk, some concerns, or high risk, according to the RoB 2 algorithm.

#### 2.4.4. Measures of Treatment Effect

Individual study effect estimates were extracted from the original reports or calculated from the reported data where necessary. Results are presented narratively using risk ratios (RRs) with 95% confidence intervals (CIs) for dichotomous outcomes and available estimates for continuous outcomes. No pooled effect estimates were calculated.

#### 2.4.5. Unit of Analysis

The individual participant was the unit of analysis.

#### 2.4.6. Dealing with Missing Data

We considered missing outcome data at the individual study level as part of the risk-of-bias assessment and took them into account when evaluating the certainty of evidence and interpreting the findings. No imputation of missing data was performed.

#### 2.4.7. Assessment of Heterogeneity

Clinical heterogeneity was assessed by comparing key study characteristics, including participant phenotype, HRF composition, comparator formula, and duration of follow-up (see included studies). Differences in participant phenotype and the definition and timing of tolerance assessment were considered to limit the clinical interpretability of pooled estimates. Because no outcome was pooled across trials, statistical heterogeneity (Chi-squared test, I^2^) was not calculated.

#### 2.4.8. Assessment of Reporting Biases

Because no outcome was pooled across trials, funnel plots and statistical tests for small-study effects were not used to assess publication bias.

#### 2.4.9. Data Synthesis and Certainty of Evidence

No outcome was pooled across trials; results are summarized narratively throughout. A subgroup analysis by CMA phenotype was planned but not performed due to insufficient data; no sensitivity analyses were performed. Certainty of the evidence was assessed using the Grading of Recommendations Assessment, Development and Evaluation (GRADE) for the prespecified primary outcome, acquisition of tolerance to cow’s milk protein, as specified in the review protocol.

## 3. Results

### 3.1. Included Studies

Four studies met the inclusion criteria [14,15,16,17]. Figure S1 shows the study selection process. Characteristics of the included and excluded trials are summarized in Tables S2 and S3, respectively. The included studies comprised one multicenter RCT [14], one single-center RCT [15], one RCT with an additional non-randomized reference arm [17], and one small pilot RCT [16]. Together, the studies enrolled infants and young children with CMA in four European countries, with recruitment occurring between 2001 and 2019.

#### 3.1.1. Study Design

The trial by Lemoine et al. [14] was conducted at six pediatric hospital clinics in Spain, France, and Belgium, between 2014 and 2019. Participants were randomly assigned to HRF or eHF; the study formulas were provided in identical packaging, and participants, families, investigators, and study staff were blinded to treatment allocation. Diagnosis of CMA was confirmed using a double-blind, placebo-controlled food challenge (DBPCFC) before initiation of the allocated formula.

The trial by Reche et al. [15] was conducted at the University Children’s Hospital La Paz in Madrid, Spain, between September 2004 and June 2006, and was open-label.

The trial by Agostoni et al. [17] was conducted at two centers in Milan, Italy, between June 2001 and December 2005, and was unblinded. Infants who discontinued breastfeeding between the fourth and sixth completed month of life were randomized to soy formula, casein hydrolysate, or HRF. A separate group that continued breastfeeding to 12 months was followed as a non-randomized reference group.

The trial by D’Auria et al. [16] was conducted at San Paolo Hospital in Milan, Italy, between March and December 2001.

#### 3.1.2. Sample Size

In Lemoine et al. [14], the sample-size calculation indicated that 53 participants per group were required. A total of 117 infants were screened, and 105 were randomized and included in the intention-to-treat analysis (50 HRF, 55 eHF). The main text reported that 71 infants completed the 12-month visit, whereas the participant flow diagram accounted for 69 infants at this visit (33 HRF and 36 eHF).

Reche et al. [15] randomized 92 infants (46 per group), of whom 81 completed the study (41 HRF, 40 eHF).

Agostoni et al. [17] reported a formal sample-size calculation, indicating that 29 participants per group were required to detect the prespecified difference in change in weight-for-age Z-score. Of 160 enrolled infants, 108 were randomized to the three formula groups (37 soy, 35 casein hydrolysate, 36 HRF), while 52 continued breastfeeding and formed a non-randomized reference group. The final analysis included 32, 31, 30, and 32 participants, respectively.

D’Auria et al. [16] randomized 16 infants, eight to HRF and eight to soy formula; all completed the study. No formal sample-size calculation was reported.

#### 3.1.3. Participants

All four studies enrolled infants with confirmed CMA, although age, phenotype, and diagnostic procedures differed.

Lemoine et al. [14] enrolled infants younger than 10 months with recently diagnosed CMA. Both IgE-mediated and non-IgE-mediated phenotypes were included; 54.0% of infants in the HRF group and 52.7% in the eHF group were cow’s-milk-specific IgE positive. Infants with anaphylactic reactions above Grade II were excluded.

Reche et al. [15] enrolled infants with IgE-mediated CMA, characterized by immediate reactions to cow’s milk protein together with evidence of specific IgE by skin prick testing or serum testing. Cow’s milk challenge was performed unless contraindicated by the clinical history.

Agostoni et al. [17] included infants with IgE-mediated CMA diagnosed within the first 6 months of life. Sensitization was assessed by skin prick testing and/or serum-specific IgE, and CMA was confirmed by DBPCFC, except in infants with a history of anaphylaxis.

D’Auria et al. [16] enrolled infants aged 6–16 months with CMA and atopic dermatitis. CMA was confirmed by DBPCFC or open challenge, as appropriate. The study did not explicitly classify the population as IgE- or non-IgE-mediated CMA.

#### 3.1.4. Interventions and Comparators

All four studies compared an HRF with at least one other specialized formula, but the HRF products themselves differed substantially. Lemoine et al. [14] and Reche et al. [15] used HRFs manufactured by Laboratorios Ordesa (Barcelona, Spain). In Lemoine et al. [14], the same formula was co-marketed under two brand names, Blemil Plus^®^ Hydrolysed Rice (Ordesa) and Modilac Expert Riz^®^ (Laboratoires Modilac, Levallois-Perret, France). The HRFs in both studies were supplemented with amino acids: lysine and tryptophan in Lemoine et al. [14] and lysine and threonine in Reche et al. [15]. Agostoni et al. [17] used a product from a different manufacturer (Risolac-2, Heinz, Milan, Italy); neither its degree of hydrolysis nor its amino acid supplementation was reported. D’Auria et al. [16] used an unbranded product, with manufacturer, hydrolysis degree, and amino acid content all unreported. Only Reche et al. [15] reported the peptide molecular weight distribution (8% of peptides > 5000 Da vs. 2.8% in the eHF comparator; 70% < 2000 Da). The comparator was eHF in Lemoine et al. [14] and Reche et al. [15], casein hydrolysate and soy formula in Agostoni et al. [17], and soy formula alone in D’Auria et al. [16].

Formula administration and follow-up both lasted 6 months in Agostoni et al. [17] and D’Auria et al. [16] and 12 months in Lemoine et al. [14]. In Reche et al. [15], follow-up extended to approximately 24 months, but formula administration varied by infant and ended once tolerance was confirmed.

#### 3.1.5. Funding

The trial by Lemoine et al. [14] was funded by Laboratoires Modilac and Laboratorios Ordesa. Both companies initiated the study, participated in data collection, and coordinated the analysis. Several authors reported employment or other financial relationships with the sponsors. Reche et al. [15] were supported by an unconditional grant from Laboratorios Ordesa, and three co-authors were employees of that company. Funding was not reported for Agostoni et al. [17] or D’Auria et al. [16].

### 3.2. Risk of Bias

Risk of bias for the primary outcome and for growth is summarized in Figure S2. For the primary outcome, both contributing trials were judged at high overall risk of bias. In Lemoine et al. [14], the OFC confirming tolerance was unblinded. Participant accounting was also inconsistent: the main text reported that 71 infants completed the final 12-month visit, whereas the supplementary flow diagram accounted for 69 participants (33 HRF and 36 eHF). In the HRF arm, the number of infants reported in the IgE subgroup analysis for tolerance acquisition did not match the number reported at baseline. These discrepancies could not be resolved from the published information.

In Reche et al. [15], the method used for allocation concealment was not described, and the trial was open-label. Tolerance was not confirmed by a standardized challenge in most infants; many were classified as still allergic based solely on clinical judgment. For growth, risk of bias was more variable across the four trials, with D’Auria et al. [16] having the most complete methodological reporting and Agostoni et al. [17] the least, due to its unblinded design and substantial, arm-specific attrition.

### 3.3. Certainty of Evidence

Using GRADE, the certainty of evidence for the primary outcome, acquisition of tolerance to cow’s milk, was very low at all assessed time points (Table S4), reflecting risk of bias and imprecision in both contributing trials.

### 3.4. Studies Awaiting Results

Two additional RCTs have evaluated a new HRF containing two manufactured human milk oligosaccharides (2’-FL and LNnT). RIGHT-HY (Zemrani et al. [8]; NCT06633289), a hypoallergenicity trial using a DBPCFC, has been completed and enrolled 43 participants, but no results have been reported. RIGHT-GO (Zemrani et al. [7]; NCT06633250), a noninferiority growth trial with a planned sample size of 104, remains listed as recruiting in the trial registry, although its estimated completion date was August 2026. No results have been reported.

### 3.5. Effects of Interventions

A summary of all outcomes is presented in Table S4. Given the small number of studies contributing to each comparison, results are summarized narratively.

#### 3.5.1. Acquisition of Tolerance to Cow’s Milk Protein

**Figure 1:**
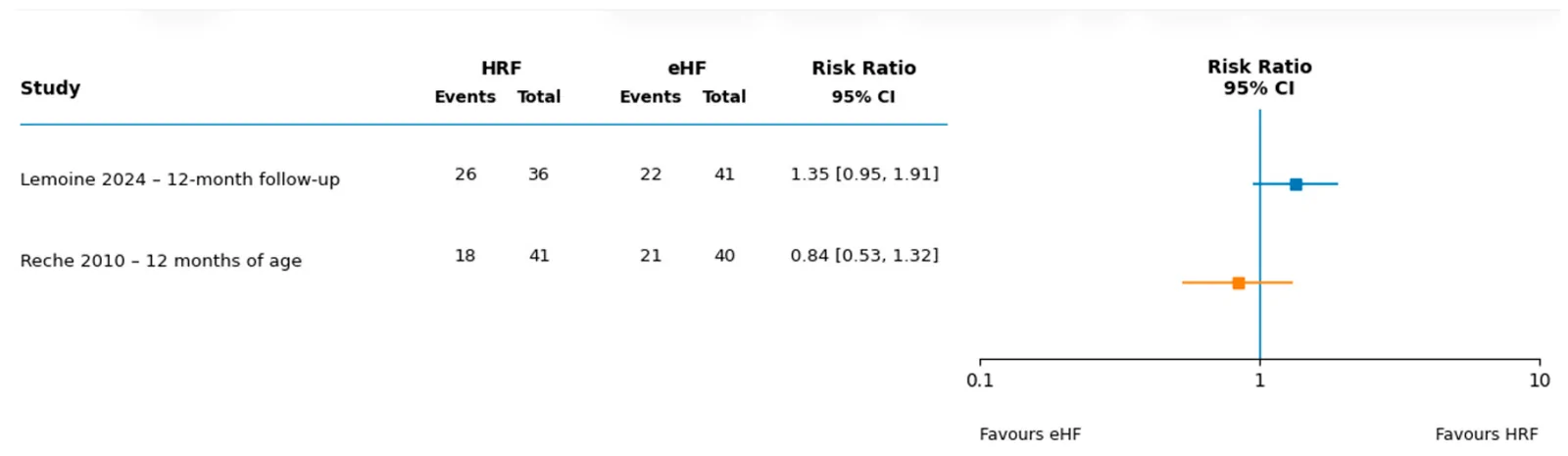
Acquisition of tolerance to cow’s milk protein: HRF versus eHF. Individual study risk ratios (RRs) with 95% confidence intervals (CIs); no pooled estimate. Reche et al. assessed tolerance at 12 months of age, whereas Lemoine et al. reported tolerance acquisition during 12 months of follow-up. HRF, hydrolyzed rice formula; eHF, extensively hydrolyzed formula.

Two studies compared HRF with eHF for this outcome (Figure 1); the eHF was casein-based in Lemoine et al. [14] and a casein-whey blend (40% casein, 60% whey) in Reche et al. [15].

Lemoine et al. [14] analyzed tolerance acquisition in 77 infants with reported age-at-tolerance data (36 HRF, 41 eHF). During the 12-month follow-up, a higher proportion of infants receiving HRF than eHF acquired tolerance to cow’s milk protein (available-case analysis: 26/36 [72.2%] vs. 22/41 [53.7%]; RR 1.35, 95% CI 0.95–1.91).

The median age at tolerance acquisition was 16.3 months in the HRF group and 20.4 months in the eHF group (*p* = 0.18). Both findings numerically favored HRF but were not statistically significant.

Reche et al. [15] (*n* = 81) reported an effect estimate in the opposite direction at 12 months of age (18/41 HRF vs. 21/40 eHF tolerant; RR 0.84, 95% CI 0.53 to 1.32) and at 24 months (31/41 vs. 31/40; RR 0.98, 95% CI 0.77 to 1.24), with no significant difference at any time point.

The two available trials yielded inconsistent results for this outcome, with clinical differences—including population (IgE-mediated only in Reche et al. [15] vs. mixed IgE- and non-IgE-mediated CMA in Lemoine et al. [14]), eHF composition (casein-only vs. casein-whey blend), and blinding status—that argue against pooling. Neither trial was powered to detect a between-group difference in tolerance acquisition, so no conclusion can be drawn regarding the effect of HRF on this outcome.

#### 3.5.2. Growth and Nutritional Outcomes

Two RCTs compared HRF with eHF [14,15]. Compared with eHF, HRF had no significant effect on weight-for-length Z-score (1 RCT, *n* = 105; group effect *p* = 0.28) or on any other anthropometric parameter assessed—weight, length, body mass index (BMI), or head circumference Z-score (all *p* ≥ 0.22) [14]. Similarly, no between-group differences were observed for weight-, length-, or weight-for-height Z-scores at any time point in Reche et al. [15] (*n* = 81); however, these data were reported only graphically, without extractable numerical values.

Two RCTs compared HRF with soy formula [16,17]. Compared with soy formula, HRF was associated with a greater increase in weight-for-age Z-score from 6 to 12 months in the unadjusted comparison (1 RCT, *n* = 62; mean difference +0.34 z; *p* = 0.019), but the difference was no longer statistically significant after Bonferroni correction for multiple comparisons (*p* = 0.090) [17]. A similar, non-significant-after-correction pattern was seen for casein hydrolysate vs. soy in the same trial (mean difference +0.32 z; *p* = 0.029 crude, *p* = 0.120 corrected). No between-group difference was found for length- or weight-for-length Z-score change (both *p* ≥ 0.286).

D’Auria et al. [16] (1 RCT, *n* = 16) found no significant difference between HRF and soy formula for any growth or biochemical parameter; however, with only eight infants per group, this trial was underpowered to detect a clinically meaningful difference.

#### 3.5.3. Safety, Tolerability, and Allergic Outcomes

Immediate allergic reactions to the study formula were rare in the two trials that assessed this outcome [14,15]. No infant assigned to HRF experienced an immediate allergic reaction, whereas one infant assigned to eHF did so in each trial (0/50 vs. 1/55 in Lemoine et al. [14]; 0/46 vs. 1/46 in Reche et al. [15]). The number of events was too small to draw conclusions about differences between formulas.

Overall and product-related adverse event rates were otherwise similar between HRF and eHF [14], and no serious adverse events were considered treatment-related in either group. In D’Auria et al. [16], no adverse reactions to the administered formulas were reported in either the HRF group or the soy formula group. However, the small sample size (*n* = 16) limited the ability to detect uncommon adverse events.

Arsenic exposure was assessed only in Lemoine et al. [14]. Total and inorganic arsenic were measured in urine and total arsenic in hair. Urinary arsenic concentrations did not differ significantly between formulas (2.24 vs. 2.30 µg/L; *p* = 0.48), although the reported values did not specify whether they represented total or inorganic arsenic.

In Agostoni et al. [17], withdrawals occurred for different reasons across treatment groups. In the HRF and casein-hydrolysate groups, withdrawals were due to poor formula acceptance, with similar rates (6/36 vs. 4/35). In the soy group, withdrawals (5/37) were due to secondary soy sensitization.

## 4. Discussion

### 4.1. Summary of Main Findings

This review aimed to evaluate evidence from RCTs on the effect of HRF on the acquisition of tolerance to cow’s milk proteins in infants and young children with confirmed CMA and, secondarily, its efficacy, safety, and tolerability, as well as its effects on growth and nutritional adequacy. Four RCTs, with sample sizes ranging from 16 to 108 participants (321 in total), met the inclusion criteria. The trials differed in HRF product, comparator, and follow-up duration.

For the primary outcome, acquisition of tolerance to cow’s milk proteins, only two trials provided data, both comparing HRF with eHF. Reche et al. assessed tolerance at 12 months of age, whereas Lemoine et al. assessed tolerance acquisition during 12 months of follow-up. Neither trial found a statistically significant difference between formulas at 12 months, and neither was powered to detect a between-group difference in this outcome. These underpowered trials cannot rule out a clinically important difference.

Regarding growth, the two trials assessing this outcome found no significant differences between HRF and eHF. In one trial, comparing HRF (and casein hydrolysate) with soy formula, both hydrolysates showed a greater gain in weight-for-age Z-score in an unadjusted comparison, but the difference was no longer significant after Bonferroni correction for multiple comparisons.

Regarding safety and tolerability, few allergic reactions to the study formulas were reported in two trials, and no clear differences in adverse events were observed between HRF and comparator formulas. Urinary arsenic concentrations, assessed in one trial, did not differ between formulas. However, these measurements do not establish long-term safety, particularly because the reported urinary concentrations were not clearly identified as total or inorganic arsenic and the available data do not permit a comprehensive assessment of exposure.

### 4.2. Quality of the Evidence

The certainty of evidence for the primary outcome was very low, reflecting risk of bias and imprecision in both contributing trials. Confidence in the growth findings was also limited by risk of bias and small sample sizes. The safety evidence was limited by the small number of participants and events and by incomplete reporting across trials; risk of bias was not formally assessed for this outcome.

### 4.3. Limitations of the Evidence

An important limitation is the heterogeneity of the HRFs evaluated, as not all hydrolyzed formulas are equivalent. Their characteristics may vary according to the hydrolysis process, the degree of hydrolysis, and the amino acid composition. Moreover, the HRFs evaluated in the included trials were incompletely characterized, particularly with regard to degree of hydrolysis and amino acid supplementation. Consequently, evidence regarding the efficacy and safety of an individual HRF should be considered product-specific and should not be automatically extrapolated to other HRFs. Differences across trials in CMA phenotype, diagnostic criteria, comparator formulas, and follow-up duration further limited comparability and the ability to draw conclusions.

Interpretation of the findings from Lemoine et al. [14] was also limited by unresolved inconsistencies in participant accounting and outcome reporting.

The applicability of the findings to children with severe CMA remains uncertain, and none of the included RCTs directly compared HRF with AAF.

### 4.4. Strengths and Limitations of the Review Process

This review has several methodological strengths. The protocol was prospectively registered, and the review followed PRISMA and Cochrane guidance. A comprehensive search without language restrictions was supplemented by trial registries, reference screening, and contact with experts. Study selection, data extraction, and risk-of-bias assessment were performed independently by at least two reviewers. Risk of bias and certainty of evidence were assessed using RoB 2 and GRADE, respectively. Restriction to RCTs provides a more internally valid, although smaller, evidence base than previous reviews that included heterogeneous study designs.

Several limitations should be acknowledged. Only four RCTs met the inclusion criteria, with small sample sizes and methodological limitations that reduced confidence in the findings. Clinical and methodological heterogeneity precluded meaningful pooling of the data. Planned subgroup analyses by CMA phenotype could not be performed because of insufficient data, and sensitivity analyses were not applicable in the absence of pooled estimates. Finally, restriction to RCTs meant that non-randomized evidence, including studies addressing tolerance acquisition, hypoallergenicity, and growth, was not included in the systematic review and was considered only as supportive evidence in the Discussion.

### 4.5. Interpretation in the Context of Other Evidence

Although our review was restricted to RCTs, several non-randomized studies provide additional evidence on tolerance acquisition, hypoallergenicity, and growth. Terracciano et al. [9], excluded because formula allocation was determined by calendar quarter of diagnosis rather than individual randomization, reported a shorter time to cow’s milk tolerance with HRF than with eHF. Two prospective non-randomized studies also evaluated tolerance acquisition [18,19]. Across these studies, tolerance rates with HRF were generally similar to those with soy formula and whey-based eHF but lower than with casein-based eHF supplemented with *Lactobacillus rhamnosus* GG (LGG); in Nocerino et al. [19], they were higher than with AAF. Interpretation is limited by non-randomized formula allocation, with potential confounding arising from temporal differences [9] and physician-directed formula selection [18,19].

Hypoallergenicity is defined as tolerance by at least 90% of children with CMA with 95% confidence [20]. Three non-randomized studies evaluated the hypoallergenicity of HRF [21,22,23], all of which reported no allergic reactions to the HRF tested. Fiocchi et al. [23] (*n* = 100) and Vandenplas et al. [21] (*n* = 40) provided sufficient evidence to meet the American Academy of Pediatrics criterion, whereas the small sample in Fiocchi et al. [22] (*n* = 18) did not. Fiocchi et al. [22,23] assessed tolerance by DBPCFC, whereas Vandenplas et al. [21] used open clinical observation. However, the studies evaluated different HRF products and differed in CMA phenotype and methods of assessing tolerance. These findings should therefore be considered product-specific rather than evidence of hypoallergenicity of HRF as a class.

Evidence from non-randomized studies was also generally reassuring regarding growth. Growth among infants receiving HRF was broadly comparable with that observed with other specialized formulas [19,21,24], although Savino et al. [24] reported lower weight-for-age Z-scores at some time points compared with healthy controls.

Additional indirect evidence comes from an RCT in healthy term infants [25], which found no differences in weight, length, or head circumference between infants receiving HRF and those receiving standard cow’s milk formula during the first 16 weeks of life.

Overall, the broader evidence does not suggest impaired growth with the HRFs studied, but its interpretation is limited by predominantly observational designs, heterogeneous populations and comparators, and differences between HRF products.

### 4.6. Earlier Systematic Reviews

Two systematic reviews have previously addressed HRF for the management of CMA. Saad et al. [3] included 17 studies comprising RCTs, quasi-RCTs, non-randomized controlled studies, and studies in both children with CMA and healthy children. The authors concluded that HRF can substitute for cow’s milk formula in the management of CMA, is well tolerated, has varying effects on growth, and shows promise in reducing atopic symptoms, while noting that variability across studies precluded meta-analysis. In contrast, our conclusions are based exclusively on evidence from four RCTs.

The World Allergy Organization Diagnosis and Rationale for Action against Cow’s Milk Allergy (DRACMA) guideline update [12] systematically reviewed RCTs and non-randomized evidence in children with CMA and found no difference in effect between HRF and eHF, or between HRF and soy formula, at very low certainty. However, its search predated publication of the most recent RCT [14].

### 4.7. Implications for Practice

Available evidence does not establish whether HRF is equivalent, superior, or inferior to eHF or soy formula for tolerance acquisition or growth in infants and children with CMA. Current guidelines nonetheless differ in their positioning of HRF: ESPGHAN [1] considers it an alternative to cow’s milk-based eHF, and WAO DRACMA [26] suggests either eHF or HRF as a first option for infants with IgE- or non-IgE-mediated CMA, albeit as a conditional recommendation based on very low-certainty evidence. In contrast, the Global Allergy and Asthma European Network (GA2LEN) [27] makes no recommendation for or against HRF due to insufficient evidence. A ‘no recommendation for or against’ does not mean the evidence shows no benefit. It means the panel had too little confidence in the evidence to recommend one option over another. In practice, this allows the choice to vary by patient, guided by shared decision-making between clinicians and families. This reflects how panels can reach different conclusions from the same limited evidence base, rather than reflecting substantive disagreement about the underlying data.

### 4.8. Implications for Research

In line with conclusions from earlier systematic reviews of specialized formula-based management of CMA, further well-designed RCTs are needed.

Future trials should be adequately powered, preferably independently funded, and designed to detect predefined clinically meaningful differences. Trials assessing superiority in tolerance acquisition should be distinguished from those assessing noninferiority in growth.

Future trials should use adequately described randomization with allocation concealment, standardized and preferably blinded outcome assessment, primarily for tolerance acquisition, analyze participants on an intention-to-treat basis, and be prospectively registered. Each HRF should be evaluated separately, given how substantially these products differ, and its composition should be adequately characterized and reported. Hypoallergenicity, not assessed in any of the included trials, should be evaluated. Participants should be clearly characterized and, where appropriate, stratified according to CMA phenotype. Future trials should prioritize clinically important outcomes, including tolerance acquisition, growth, and safety. Newer HRF formulations, including those containing human milk oligosaccharides, are awaiting results (see Section 3.4) and have not yet yielded publicly available results.

## 5. Conclusions

Current RCT evidence is insufficient to establish whether HRF is equivalent, superior, or inferior to eHF or soy formula for tolerance acquisition or growth in infants and young children with CMA. The certainty of evidence for tolerance acquisition is very low. The available trials did not identify clear growth or safety concerns, but their small sample sizes and methodological limitations preclude firm conclusions. Adequately powered, product-specific RCTs using standardized outcome definitions are needed.

## Data Availability

The original contributions presented in this study are included in the article/supplementary material. Further inquiries can be directed to the corresponding author.

## Supplementary Materials

The following supporting information can be downloaded at: https://www.mdpi.com/article/10.3390/nu18203429/s1, Figure S1: PRISMA flow diagram; Figure S2: Risk-of-bias assessment; Table S1: Search strategy; Table S2: Characteristics of the included trials (Excel file); Table S3: Characteristics of the excluded studies with reasons; Table S4: GRADE evidence profile: acquisition of tolerance to cow’s milk protein (primary outcome); Table S5: PRISMA 2020 checklist.
