# Supplementary material for: Hydrolyzed Rice Formula for the Management of Cow’s Milk Allergy in Infants and Young Children: A Systematic Review of Randomized Controlled Trials
Source: Nutrients. 2026 Oct 19;18(20):3429. doi: 10.3390/nu18203429 (PMC13708501; doi:10.3390/nu18203429)
Supplement: Supplementary file 1 [file nutrients-18-03429-s001.zip › Figure_S1_PRISMA_Flow_diagram.pdf]

Figure S1. PRISMA flow diagram

PRISMA 2020 flow diagram for new systematic reviews which included searches of databases, registers and other sources

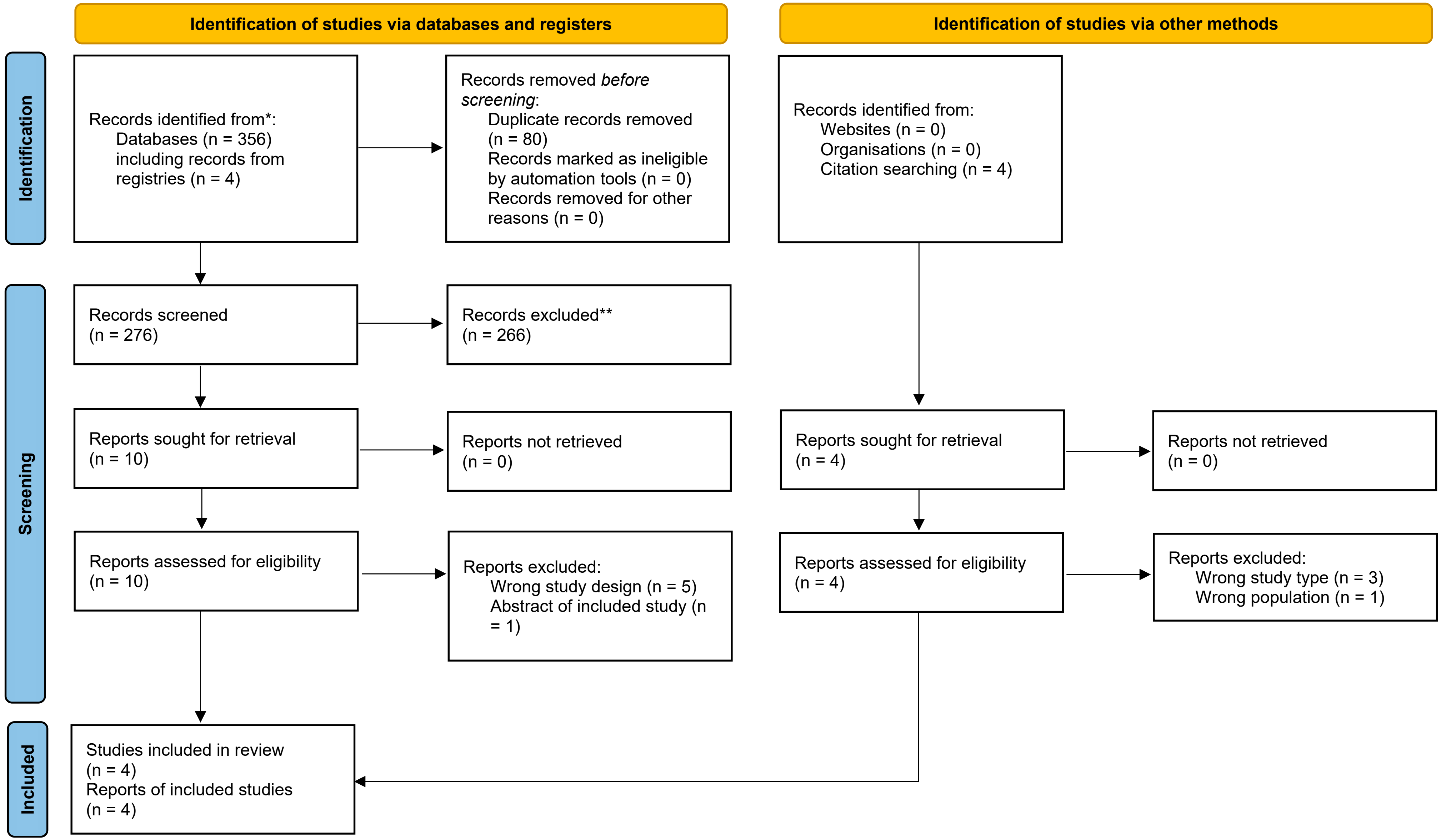

*Consider, if feasible to do so, reporting the number of records identified from each database or register searched (rather than the total number across all databases/registers).
**If automation tools were used, indicate how many records were excluded by a human and how many were excluded by automation tools.

Figure S1. PRISMA flow diagram

PRISMA 2020 flow diagram for new systematic reviews which included searches of databases, registers and other sources

Source: Page MJ, et al. BMJ 2021;372:n71. doi: 10.1136/bmj.n71.

This work is licensed under CC BY 4.0. To view a copy of this license, visit https://creativecommons.org/licenses/by/4.0/
