# Supplementary material for: Hydrolyzed Rice Formula for the Management of Cow’s Milk Allergy in Infants and Young Children: A Systematic Review of Randomized Controlled Trials
Source: Nutrients. 2026 Oct 19;18(20):3429. doi: 10.3390/nu18203429 (PMC13708501; doi:10.3390/nu18203429)
Supplement: Supplementary file 1 [file nutrients-18-03429-s001.zip › Table_S1_search strategy.pdf]

Search strategy

1. MEDLINE (Pubmed)

22	(((((((((Pediatrics[MeSH Terms]) OR (((pediatric*) OR (peadiatric*)) OR (paediatric*))) OR (child[MeSH Terms])) OR (infant[MeSH Terms])) OR (((("infancy"[All Fields]) OR ("new born"[All Fields])) OR ("new borns"[All Fields])) OR ("newly born"[All Fields]))) OR ((infant*) OR (newborn*))) OR (perinat* OR neonat* OR baby* OR toddler* OR minor* OR child* OR prematur* OR preterm*)) OR ((((((((((("baby"[All Fields])) OR ("babies"[All Fields])) OR ("babe"[All Fields])) OR ("babes"[All Fields]))) OR ("kid"[All Fields])) OR ("kids"[All Fields])) OR ("premature"[All Fields])) OR ("pre mature"[All Fields])) OR ("pre matures"[All Fields])) OR ("prematures"[All Fields])) OR ("prematurity"[All Fields])) OR ("pre maturity"[All Fields]))) OR ((("boy"[All Fields]) OR ("boys"[All Fields])) OR (girl*))) AND (((("Milk Hypersensitivity"[Mesh]) OR ((((("milk allergy"[All Fields]) OR ("milk protein allergy"[All Fields])) OR ("milk sensitivity"[All Fields])) OR ("milk hypersensitivity"[All Fields])) OR ("cma"[All Fields]))) OR ((("allergic reaction to cow s milk") OR ("allergic reaction to milk")) OR ("allergy to milk"[All Fields]))) OR ((((((("milk protein allergy"[All Fields]) OR ("dairy allergy"[All Fields]))) OR ("dairy allergies"[All Fields])) OR ("milk allergies"[All Fields])) OR ("milk hypersensitivities"[All Fields])) OR ("bovine milk allergy"[All Fields]) OR ("milk allergic reaction"[All Fields]) OR ("milk protein hypersensitivity"[All Fields])))) AND (((((rice AND (hydrolysate OR hydrolyzed OR hydrolysed OR hydroly*)) OR (rice AND (formula OR formulas))) OR ('rice peptides' OR 'rice protein peptides')) OR (hrf OR hrfs OR rhf)) OR ('rice protein' OR 'rice proteins'))
21	((((rice AND (hydrolysate OR hydrolyzed OR hydrolysed OR hydroly*)) OR (rice AND (formula OR formulas))) OR ('rice peptides' OR 'rice protein peptides')) OR (hrf OR hrfs OR rhf)) OR ('rice protein' OR 'rice proteins')
20	'rice protein' OR 'rice proteins'
19	hrf OR hrfs OR rhf
18	'rice peptides' OR 'rice protein peptides'
17	rice AND (formula OR formulas)
16	rice AND (hydrolysate OR hydrolyzed OR hydrolysed OR hydroly*)
15	((("Milk Hypersensitivity"[Mesh]) OR ((((("milk allergy"[All Fields]) OR ("milk protein allergy"[All Fields])) OR ("milk sensitivity"[All Fields])) OR ("milk hypersensitivity"[All Fields])) OR ("cma"[All Fields]))) OR ((("allergic reaction to cow s milk") OR ("allergic reaction to milk")) OR ("allergy to milk"[All Fields]))) OR ((((((("milk protein allergy"[All Fields]) OR ("dairy allergy"[All Fields]))) OR ("dairy allergies"[All Fields])) OR ("milk allergies"[All Fields])) OR ("milk hypersensitivities"[All Fields])) OR ("bovine milk allergy"[All Fields]) OR ("milk allergic reaction"[All Fields]) OR ("milk protein hypersensitivity"[All Fields]))
14	(((((("milk protein allergy"[All Fields]) OR ("dairy allergy"[All Fields]))) OR ("dairy allergies"[All Fields])) OR ("milk allergies"[All Fields])) OR ("milk hypersensitivities"[All Fields])) OR ("bovine milk allergy"[All Fields]) OR ("milk allergic reaction"[All Fields]) OR ("milk protein hypersensitivity"[All Fields])
13	(("allergic reaction to cow s milk") OR ("allergic reaction to milk")) OR ("allergy to milk"[All Fields])
12	(((("milk allergy"[All Fields]) OR ("milk protein allergy"[All Fields])) OR ("milk sensitivity"[All Fields])) OR ("milk hypersensitivity"[All Fields])) OR ("cma"[All Fields])
11	"Milk Hypersensitivity"[Mesh]
10	(((((((Pediatrics[MeSH Terms]) OR (((pediatric*) OR (peadiatric*)) OR (paediatric*))) OR (child[MeSH Terms])) OR (infant[MeSH Terms])) OR (((("infancy"[All Fields]) OR ("new born"[All Fields])) OR ("new borns"[All Fields])) OR ("newly born"[All Fields]))) OR ((infant*) OR (newborn*))) OR (perinat* OR neonat* OR baby* OR toddler* OR minor* OR child* OR prematur* OR preterm*)) OR ((((((((((("baby"[All Fields])) OR ("babies"[All Fields])) OR ("babe"[All Fields])) OR ("babes"[All Fields]))) OR ("kid"[All Fields])) OR ("kids"[All Fields])) OR ("premature"[All Fields])) OR ("pre mature"[All Fields])) OR ("pre matures"[All Fields])) OR ("prematures"[All Fields])) OR ("prematurity"[All Fields])) OR ("pre maturity"[All Fields]))) OR ((("boy"[All Fields]) OR ("boys"[All Fields])) OR (girl*))
9	(("boy"[All Fields]) OR ("boys"[All Fields])) OR (girl*)

8	(((((((((((("baby"[All Fields])) OR ("babies"[All Fields])) OR ("babe"[All Fields])) OR ("babes"[All Fields]))) OR ("kid"[All Fields])) OR ("kids"[All Fields])) OR ("premature"[All Fields])) OR ("pre mature"[All Fields])) OR ("pre matures"[All Fields])) OR ("prematures"[All Fields])) OR ("prematurity"[All Fields])) OR ("pre maturity"[All Fields])
7	perinat* OR neonat* OR baby* OR toddler* OR minor* OR child* OR prematur* OR preterm*
6	(infant*) OR (newborn*)
5	((("infancy"[All Fields]) OR ("new born"[All Fields])) OR ("new borns"[All Fields])) OR ("newly born"[All Fields])
4	infant[MeSH Terms]
3	child[MeSH Terms]
2	((pediatric*) OR (peadiatric*)) OR (paediatric*)
1	Pediatrics[MeSH Terms]

2. Cochrane Central Register of Controlled Trials (CENTRAL, the Cochrane Library)

ID	Search
#1	MeSH descriptor: [Child] explode all trees
#2	MeSH descriptor: [Pediatrics] explode all trees
#3	(pediatric*) OR (peadiatric*) OR (paediatric*)
#4	(boy) OR (boys) OR (boyhood)OR (girl*)
#5	(kid) OR (kids)
#6	MeSH descriptor: [Infant] explode all trees
#7	(infant*) OR (infancy)
#8	(newborn*) OR ("new born") OR ("new-borns") OR (neonat*) OR ("newly born")
#9	(premature) OR (prematurity) OR (preterm*) OR ("pre-term") OR (prematur*)
#10	(perinat*) OR (neonat*) OR (baby) OR (baby*) OR (babies)
#11	(babe) OR (babes) OR (toddler*) OR (minor*) OR (child*)
#12	(prematures) OR ("pre-mature") OR ("pre-matures") OR ("pre-maturity")
#13	{OR #1-#12}
#14	MeSH descriptor: [Milk Hypersensitivity] explode all trees
#15	("milk allergy") OR ("milk protein allergy") OR ("milk sensitivity") OR ("milk hypersensitivity") OR ("cma")
#16	("allergic reaction to cow's milk") OR ("allergic reaction to milk") OR ("allergy to milk")
#17	("milk protein allergy") OR ("dairy allergy") OR ("milk allergies") OR ("milk protein hypersensitivity")
#18	{OR #14-#17}
#19	rice AND (hydrolysate OR hydrolyzed OR hydrolysed OR hydroly*)
#20	rice AND (formula OR formulas)
#21	("rice peptides") OR ("rice protein peptides")
#22	(hrf) OR (hrfs) OR (rhf)
#23	("'rice protein") OR ("rice proteins")
#24	{OR #19-#23}
#25	#13 AND #18 AND #24 in Trials

3. EMBASE

ID	Search
#17	#5 AND #11 AND #16
**#16**	**#12 OR #13 OR #14 OR #15**

#15	boy OR boys OR boyhood OR girl*
#14	'pediatrics'/exp OR pediatric* OR peadiatric* OR paediatric*
#13	child'/exp OR perinat* OR neonat* OR baby OR baby* OR babies OR babe OR babes OR toddler* OR minor* OR kid OR kids OR child* OR prematur* OR premature OR pre-mature OR pre-matures OR prematures OR prematurity OR pre-maturity OR preterm*
#12	'infant'/exp OR 'infant*' OR 'infancy' OR 'newborn*' OR 'new born' OR 'new borns' OR 'newly born'
**#11**	**#6 OR #7 OR #8 OR #9 OR #10**
#10	rice protein' OR 'rice proteins'
#9	hrf OR hrfs OR rhf
#8	rice peptides' OR 'rice protein peptides'
#7	rice AND (formula OR formulas)
#6	rice AND (hydrolysate OR hydrolyzed OR hydrolysed OR hydroly*)
**#5**	**#1 OR #2 OR #3 OR #4**
#4	milk protein allergy' OR 'dairy allergy' OR 'dairy allergies' OR 'milk allergies' OR 'milk hypersensitivities' OR 'bovine milk allergy' OR 'milk allergic reaction' OR 'milk protein hypersensitivity'
#3	allergic reaction to cow s milk' OR 'allergic reaction to milk' OR 'allergy to milk'
#2	milk allergy' OR 'milk protein allergy' OR 'milk sensitivity' OR 'milk hypersensitivity' OR 'cma'
#1	milk allergy'/exp
