# Supplementary material for: Hydrolyzed Rice Formula for the Management of Cow’s Milk Allergy in Infants and Young Children: A Systematic Review of Randomized Controlled Trials
Source: Nutrients. 2026 Oct 19;18(20):3429. doi: 10.3390/nu18203429 (PMC13708501; doi:10.3390/nu18203429)
Supplement: Supplementary file 1 [file nutrients-18-03429-s001.zip › Table_S3_Characteristics of the excluded studies with reason.pdf]

Table S3: Characteristics of excluded studies with reasons

First author, year	Reason for exclusion
1. Berni Canani 2013 (1)	Wrong study design: a cohort study
2. Bocquet 2019 (2)	Wrong study type: a review
3. D'Auria 2016 (3)	Conference abstract
4. Fiocchi 2006 (4)	Wrong study design: non-RCT
5. Lasekan 2006 (5)	Wrong population: healthy infants
6. Nieto-Garcia 2023 (6)	Abstract of the GRITO study: a full paper was included
7. Savino 2005 (7)	Wrong study design: a cohort study
8. Solar 2016 (8)	Abstract of the JUNGLO study – only one group reported (part of RCT)
9. Terracciano (9)	Wrong study design: quasi RCT
10. Vandenplas 2014 (10)	Wrong study design: Lack of control group

References:

1. Berni Canani R, Nocerino R, Terrin G, Frediani T, Lucarelli S, Cosenza L, et al. Formula selection for management of children with cow's milk allergy influences the rate of acquisition of tolerance: A prospective multicenter study. Journal of Pediatrics. 2013;163(3):771-7.e1.
2. Bocquet A, Dupont C, Chouraqui JP, Darmaun D, Feillet F, Frelut ML, et al. Efficacy and safety of hydrolyzed rice-protein formulas for the treatment of cow's milk protein allergy. Archives de Pediatrie. 2019;26(4):238-46.
3. D'Auria E, Pietra B, Mandelli M, Radaelli G, Verduci E, Banderali G. Effect of different hydrolyzed formulas and soy formula on tolerance acquisition in children with cow's milk allergy. Journal of Pediatric Gastroenterology and Nutrition. 2016;62:728.
4. Fiocchi A, Restani P, Bernardini R, Lucarelli S, Lombardi G, Magazzù G, et al. A hydrolysed rice-based formula is tolerated by children with cow's milk allergy: a multi-centre study. Clinical and experimental allergy : journal of the British Society for Allergy and Clinical Immunology. 2006;36(3):311-6.
5. Lasekan JB, Koo WW, Walters J, Neylan M, Luebbers S. Growth, tolerance and biochemical measures in healthy infants fed a partially hydrolyzed rice protein-based formula: a randomized, blinded, prospective trial. J Am Coll Nutr. 2006;25(1):12-9.
6. Nieto-Garcia A, Navas-López V, Espin B, Mazon A, Salhi H, Salamouras D, et al. HYDROLYZED RICE PROTEIN VERSUS EXTENSIVELY HYDROLYZED PROTEIN FORMULAS IN GROWTH AND TOLERANCE ACQUISITION OF INFANTS WITH COW'S MILK PROTEIN ALLERGY: THE GRITO STUDY. Journal of Pediatric Gastroenterology and Nutrition. 2023;76:963.
7. Savino F, Castagno E, Monti G, Serraino P, Peltran A, Oggero R, et al. Z-score of weight for age of infants with atopic dermatitis and cow's milk allergy fed with a rice-hydrolysate formula during the first two years of life. Acta paediatrica (Oslo, Norway : 1992) Supplement. 2005;94(449):115-9.
8. Solar A, Ibanez MD, Maldonado J, Nevot S, Alonso E, Plaza AM, et al. Tolerance and efficacy of extensively hydrolyzed formula for infants with IgE-mediated cow's milk

Protein Allergy (CMPA) JUNGLO study-PART 2 : extensively hydrolyzed rice protein formula. Journal of pediatric gastroenterology and nutrition. 2016;62:839-40.
9. Terracciano L, Bouygue GR, Sarratud T, Veglia F, Martelli A, Fiocchi A. Impact of dietary regimen on the duration of cow's milk allergy: A random allocation study. Clinical and Experimental Allergy. 2010;40(4):637-42.
10. Vandenplas Y, De Greef E, Hauser B. Safety and tolerance of a new extensively hydrolyzed rice protein-based formula in the management of infants with cow's milk protein allergy. European Journal of Pediatrics. 2014;173(9):1209-16.
