# Supplementary material for: Hydrolyzed Rice Formula for the Management of Cow’s Milk Allergy in Infants and Young Children: A Systematic Review of Randomized Controlled Trials
Source: Nutrients. 2026 Oct 19;18(20):3429. doi: 10.3390/nu18203429 (PMC13708501; doi:10.3390/nu18203429)
Supplement: Supplementary file 1 [file nutrients-18-03429-s001.zip › Table_S4_GRADE evidence profile- acquisition of tolerance to cow’s milk protein (primary outcome)..pdf]

Table S4. GRADE evidence profile: acquisition of tolerance to cow's milk protein (primary outcome).

Certainty assessment							№ of patients		Effect		Certainty	Importance
№ of studies	Study design	Risk of bias	Inconsistency	Indirectness	Imprecision	Other considerations	hydrolysed rice-formula	extensively hydrolysed cow's milk formula	Relative (95% CI)	Absolute (95% CI)		
**acquisition of tolerance to cow's milk (assessed at 12 months of age with; assessed by oral food challenge)**												
1<br><br>Reche 2010	randomised trials	serious[a]	not serious	not serious	very serious[b]	none	18/41 (43.9%)	21/40 (52.5%)	**RR 0.84**<br>(0.53 to 1.32)	**84 fewer per 1000**<br>(from 247 fewer to 168 more)	⨁◯◯◯<br>Very low[a,b]	CRITICAL
**acquisition of tolerance to cow's milk (during 12 months follow-up; assessed by oral food challenge)**												
1<br><br>Lemoine 2024	randomised trials	serious[c]	not serious	not serious	very serious[b]	none	26/36 (72.2%)	22/41 (53.7%)	**RR 1.35**<br>(0.95 to 1.91)	**188 more per 1000**<br>(from 27 to 488 more)	⨁◯◯◯<br>Very low[b,c]	CRITICAL

CI: confidence interval; **RR:** risk ratio

Explanations

a. Downgraded one level for risk of bias due to methodological limitations of the open-label trial and incomplete outcome data.

b. Downgraded two levels for imprecision because the sample size was small and the 95% CI was very wide, including both potentially important benefit and harm.

c. Downgraded one level for risk of bias because of concerns related to missing outcome data and outcome reporting.

GRADEpro GDT (McMaster University and Evidence Prime, Hamilton, ON, Canada; accessed [9 Sept 2026])
